# Recalcitrant primary cutaneous Rosai‐Dorfman disease. Efficacy of sirolimus and intralesional methylprednisolone

**DOI:** 10.1002/ski2.273

**Published:** 2023-08-07

**Authors:** Andrés Tirado‐Sánchez

**Affiliations:** ^1^ Hospital General de Zona 30 Instituto Mexicano del Seguro Social Mexico Mexico

## Abstract

Sinus histiocytosis or Rosai‐Dorfman disease (RDD) is a rare disorder with severe lymphadenopathy and a limited clinical course, the aetiology of which is still controversial. The disease usually affects cervical nodes, with fever, polyclonal gammopathy, and leucocytosis with neutrophilia. Pure cutaneous involvement occurs as the only manifestation in only 3% of cases. Cutaneous RDD is often associated with infections, immunodeficiency, and autoimmune disorders. A 52‐year‐old patient presented with disseminated, recurrent, and relapsed pure cutaneous RDD that responded well to treatment with sirolimus and local infiltrations of methylprednisolone. The patient had multiple nonpainful nodular and tumour‐like lesions, histiocytic infiltrates with emperipolesis were observed on histologic examination, and positive immunohistochemistry for CD68, and S100. There is no standardised treatment, then the patient was treated with various therapies, including systemic steroids, chemotherapy (cyclophosphamide, doxorubicin, vincristine, and prednisone), radiotherapy, and other immunosuppressive treatments. Some lesions were treated surgically, resulting in recurrence. Sirolimus and local infiltration with methylprednisolone were tried as salvage treatments, the patient responded well, reducing the incidence of new lesions during follow‐up, and the size of the preexisting lesions.

## Graphical abstract

Rosai‐Dorfman disease is a sinus histiocytosis with massive lymphadenopathy. Purely‐cutaneous disease is rare. There is a lack of guidelines to treat the disease and often is based in disease severity.

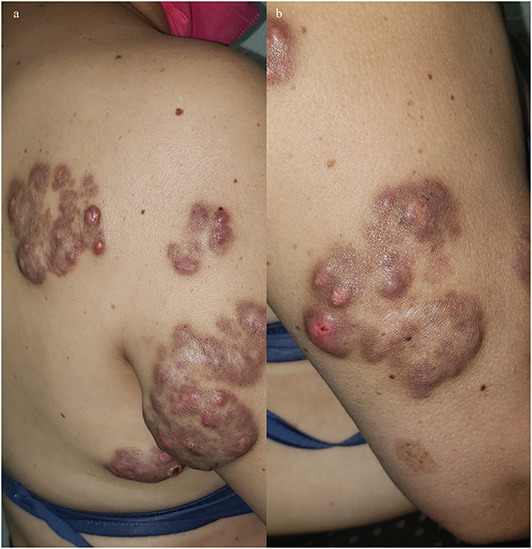

## 1 INTRODUCTION

Rosai‐Dorfman disease (RDD) is a non‐Langerhans histiocytosis that classically develops severe lymphadenopathy.^1^ Extranodal localization is common and skin involvement may reveal the disease, but diagnosis is often complicated and delayed.^2^ RDD lesions are diverse representing a diagnostic challenge. The disease is confirmed by histopathologic examination showing histiocytic infiltrates with emperipolesis. Cutaneous lesions in RDD are often self‐limited and benign, and thus, aggressive treatments are not necessary. In extensive, recalcitrant or recurrent RDD, a wide range of therapeutic options are available, including surgical intervention, topical and oral steroids, chemotherapy, often with unsatisfactory results,^3^ as observed in this case.

This case report presents a patient with disseminated and recurrent cutaneous RDD, who was treated with various treatment options without response or rapid relapse. The final episode of the disease was treated with sirolimus and intralesional steroids, limiting the appearance of new lesions and regressed preexisting manifestations. To our knowledge, this is the first report of pure cutaneous RDD treated with sirolimus.

## 2 CASE REPORT

**FIGURE 1:**
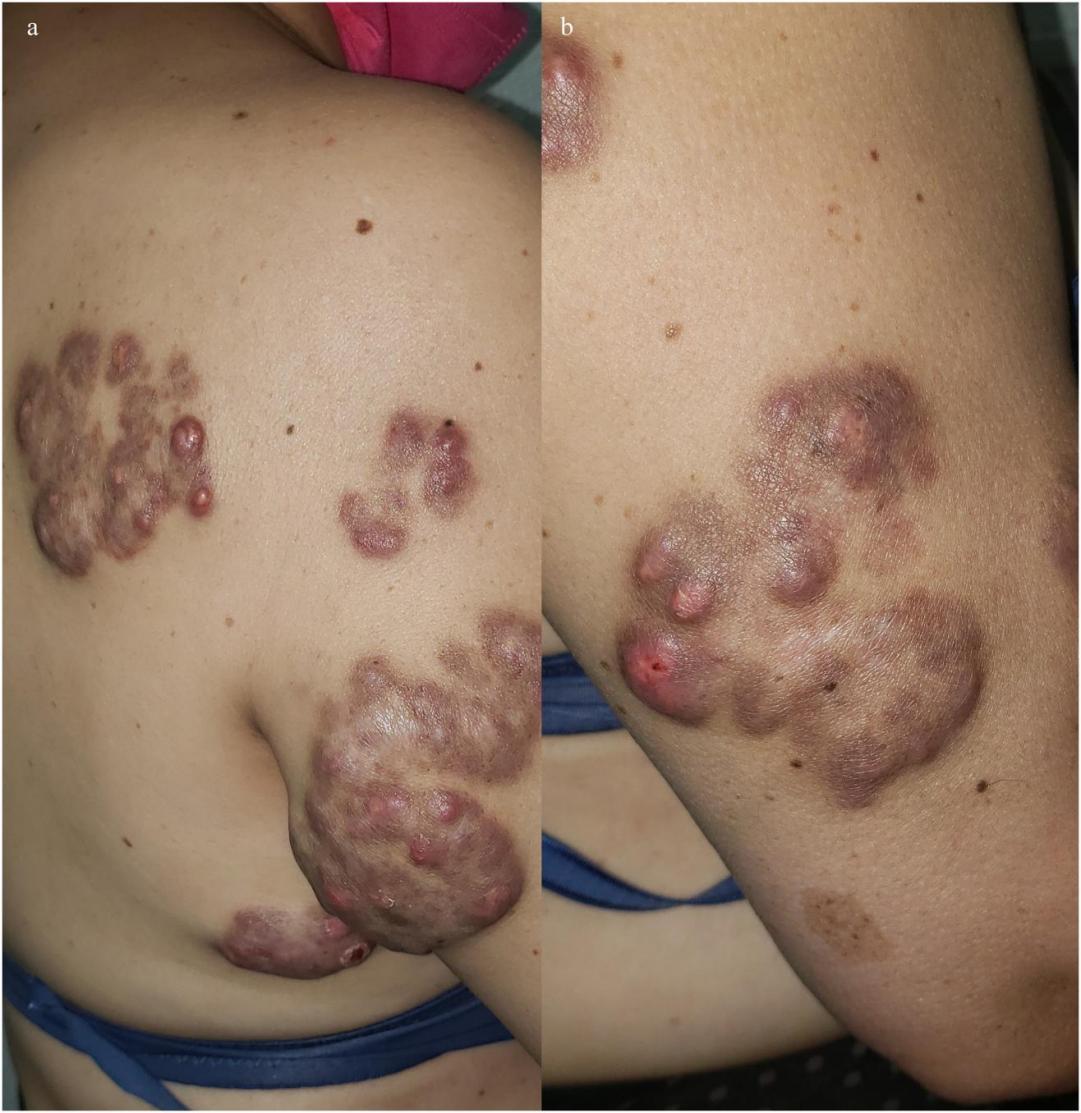
Orange‐to‐dark brown papuloerythematous nodules that coalesced into patches and affected the trunk (a) and extremities (b).

**FIGURE 2:**
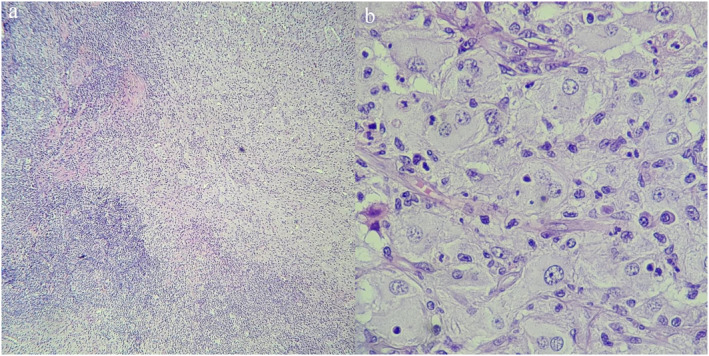
(a) Histological cutting with haematoxylin and eosin (H&E) observing diffuse inflammatory infiltrate with foamy histiocytes, ×40. (b) In the dermis, histiocytes with abundant cytoplasm and presence of emperipolesis are observed. H&E, ×100.

**FIGURE 3:**
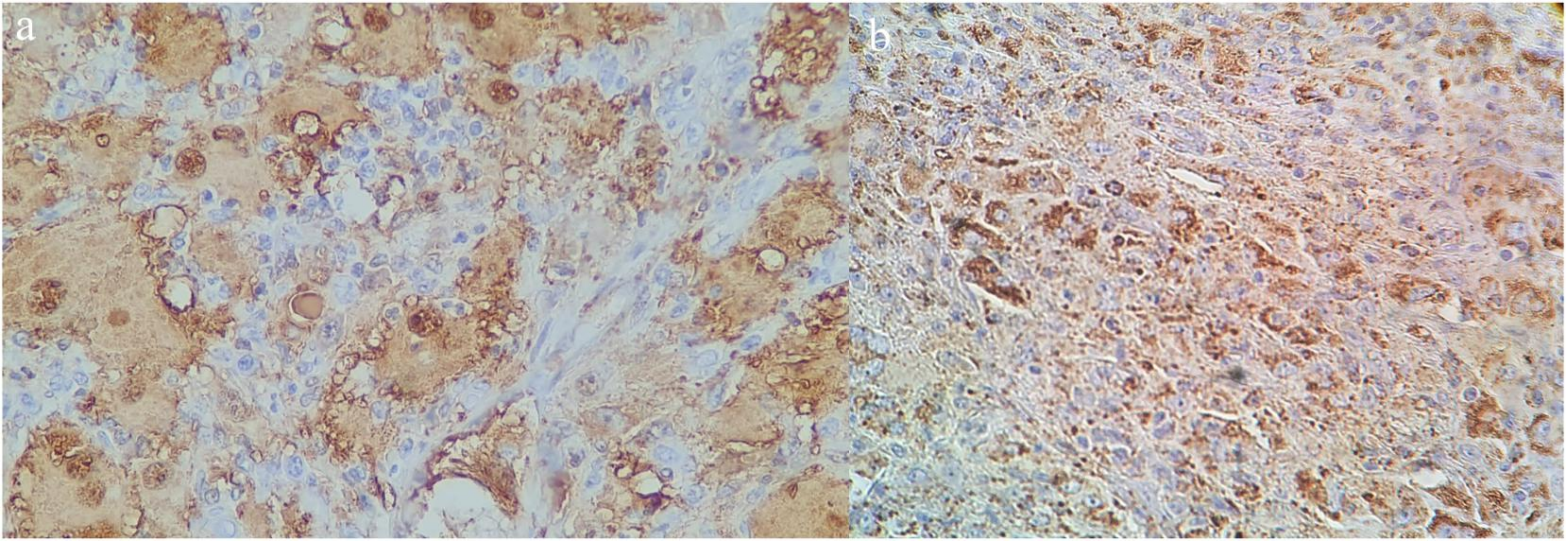
(a) Positive immunohistochemistry for S‐100 protein and (b) CD68, ×100.

A 56‐year‐old female patient with type 2 diabetes presented to our practice with multiple orange to dark brown papuloerythematous nodules and tumour‐like appearance without lymphadenopathy and involved the legs, arms, and trunk (Figure 1). The lesions were asymptomatic and asymmetrically arranged. Five years elapsed between the appearance of the skin lesions and diagnosis. The patient had no neurologic, respiratory, or ocular impairment. Histologically, there was a dense dermal infiltrate of large histiocytic cells with pale eosinophilic cytoplasm. The histiocytes had neutrophils and lymphocytes in the cytoplasm (emperipolesis) surrounded by lymphocytes, plasma cells, and fibrosis (Figure 2). Immunohistochemical examination showed expression of S100 and CD68 (Figure 3); CD1a and Langerine were negative, also BRAF and KRAF mutations. No abnormalities were detected on routine laboratories and computed tomography, therefore, RDD was considered purely cutaneous.

**FIGURE 4:**
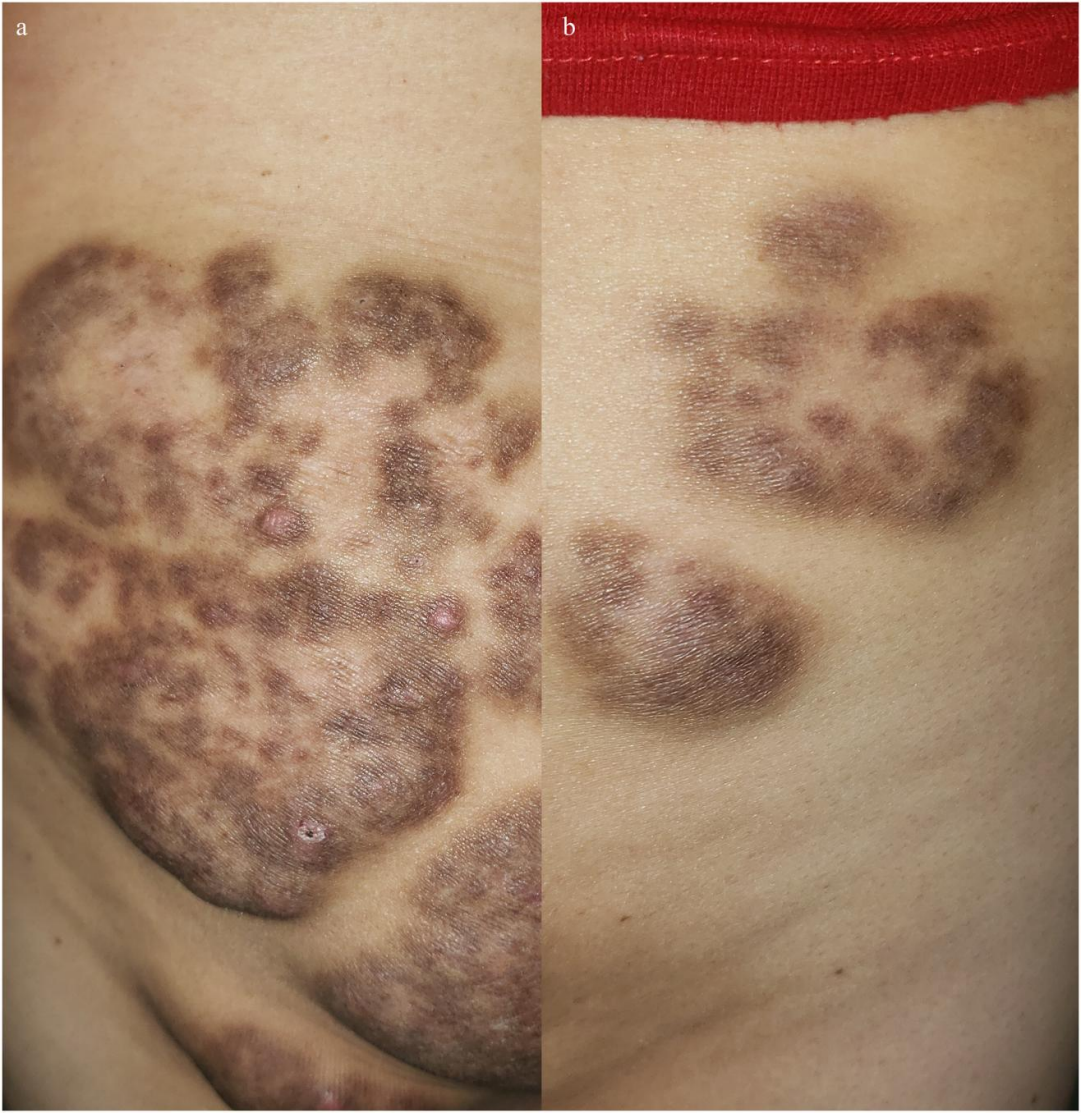
(a) Regression of some trunk lesions with sirolimus. (b) Decreased elevation of pre‐existing lesions with infiltration of methylprednisolone.

The treatments offered varied depending on the location. Due to functional impairment, the lesions were removed from the buttocks, which recurred 2 months later. She was irradiated with 30 Gy (1 cm irradiation field) in 15 sessions. The disease recurred 6–8 months later. Other previous therapies included systemic steroids with thalidomide followed by methotrexate and intralesional glucocorticoids with favourable cosmetic results in isolated lesions. Treatment with sirolimus was started at an initial dose of 3 mg per day and maintained at 1 mg daily for 6 months, with new lesions treated monthly with intralesional methylprednisolone. Sirolimus and local infiltration with methylprednisolone were tried as salvage therapy, which responded well (Figure 4), reduced the incidence of new lesions during follow‐up, and reduced the volume of preexisting lesions.

## 3 DISCUSSION

Pure cutaneous RDD is rare and easily misdiagnosed.^1^ It is an extranodal variant that occurs in only 3% of patients with this histiocytosis, with no predisposition to age or gender, in contrast to the systemic form, more common in women (2:1) in the fourth decade of life.^4^

The etiopathogenesis is unknown. It is thought to be related to dysregulation of the immune system by malignancies such as lymphoma, leukaemia, viral infections (Epstein‐Barr virus, herpes simplex virus, human herpes virus type 6, cytomegalovirus, and parvovirus B19), Bacteria such as *Brucella*, *Klebsiella rhinoscleromatis*, and *Nocardia* or immunologic diseases as triggers, HIV infection, and zoster‐related scars, previous surgery, and pneumococcal vaccination.^5^

The possibility that RDD is a neoplasm has been considered because of its relationship to histiocyte monoclonality. Immunohistochemistry of the disease shows histiocyte‐associated markers (CD68, CD163) and negative Langerhans cell markers (CD1a, CD207).^3^

Other associated markers include S100 protein and mutations in NRAS, KRAS, MAP2K1, and ARAF, suggesting a clonal origin of the disease.^6^ Mutations in mTOR, KMT2D, and NOTCH1 may also be involved in the development of the disease.^7^

The present case shows nodular and tumour‐like lesions, accounting for 79.5% and 7.7% of the cutaneous lesions in RDD according a previous study.^8^ Plaque type lesions were not observed.

Differential diagnosis of cutaneous RDD includes other histiocytoses (xanthogranuloma, reticulohistiocytoma, Langerhans cell histiocytosis), sarcoidosis, Langerhans cell histiocytosis, chronic infections (leprosy, lupus vulgaris), granuloma annulare, xanthoma, lymphoproliferative processes, particularly lymphoma.^9^ Most of these diagnoses can be ruled out with histopathology and immunohistochemical profile, displaying emperipolesis and positive S100 and CD68, and negative CD1a and Langerine, respectively.^5^, ^8^ The absence of lymph node involvement confirm cutaneous RDD.^8^

Chronic infectious diseases such as cutaneous tuberculosis, leprosy and leishmaniasis can be distinguished from ERD by the presence of caseating granulomas, the presence of vacuolated macrophages (Virchow cells) and extensive inflammatory infiltration with the presence of plasma and amastigote cells within histiocytes.^10^ In cases where diagnosis is difficult, PCR studies are required to identify the causative agent. Sarcoidosis can be distinguished from ERD by the presence of noncaseating granulomas on histopathology.^9^ In the case of xanthogranulomas, differential diagnosis with ERD is difficult because both emperipolesis and expression of S100 have been reported.^11^ However, the presence of Touton‐type giant cells and the presence of the factor XIII may help to distinguish xanthogranulomas from ERD. In Langerhans cell histiocytosis, it may manifest clinically as ERD, with S100 protein expressed in both diseases, although CD1A and Langerine are generally negative in ERD. Emperipolesis may also occur in malignant histiocytosis.^10^

In the absence of visceral involvement, therapeutic options depend on the impact of the skin lesions and include intralesional corticosteroid injections (isolated lesions),^12^ cryotherapy, radiation therapy, and surgery. For refractory or extensive disease, high‐dose thalidomide, oral dapsone, low‐dose methotrexate, oral retinoids (isotretinoin, acitretin), imatinib, and systemic steroids are used.^13^, ^14^, ^15^

Sirolimus is an understudied option for the treatment of RDD.^16^ Cooper et al^17^ first reported the use and efficacy of sirolimus in the treatment of systemic RDD with disease unresponsive to multiple treatments. This case was followed by two case reports of the efficacy of this calcineurin inhibitor in RDD lymphadenopathy,^16^ although the efficacy of sirolimus in purely cutaneous RDD has not been previously reported.

Sirolimus inhibits possible deregulation of the mTOR pathway, thereby impairing the normal development of histiocyte progenitor cells, resulting in a reduction of these cells.^16^

Understanding the pathogenesis of histiocytosis and its clinical manifestations will allow therapeutic guidelines to be developed to reduce the consequences and severity of these diseases.

## CONFLICT OF INTEREST STATEMENT

None to declare.

## AUTHOR CONTRIBUTIONS

**Andrés Tirado‐Sánchez**: Conceptualization (lead); investigation (lead); methodology (lead); resources (lead); validation (lead); visualization (lead); writing – original draft (lead); writing – review & editing (lead).

## ETHICS STATEMENT

Not applicable.

## Data Availability

The data that support the findings of this study are available from the corresponding author upon reasonable request.
